# Allergenicity of Deamidated and/or Peptide-Bond-Hydrolyzed Wheat Gliadin by Transdermal Administration

**DOI:** 10.3390/foods9050635

**Published:** 2020-05-15

**Authors:** Ryosuke Abe, Narumi Matsukaze, Hayato Kobayashi, Yusuke Yamaguchi, Harumi Uto-Kondo, Hitoshi Kumagai, Hitomi Kumagai

**Affiliations:** 1 Department of Chemistry and Life Science, Nihon University, 1866 Kameino, Fujisawa-shi, Kanagawa 252-0880, Japan; r.abe0615@gmail.com (R.A.); mtkz74nrm1105@gmail.com (N.M.); brha19012@g.nihon-u.ac.jp (H.K.); yamaguchi.yusuke@nihon-u.ac.jp (Y.Y.); 2 Department of Bioscience in Daily Life, Nihon University, 1866 Kameino, Fujisawa-shi, Kanagawa 252-0880, Japan; kondou.harumi@nihon-u.ac.jp; 3 Department of Food Science and Nutrition, Kyoritsu Women’s University, 2-2-1 Hitotsubashi, Chiyoda-ku, Tokyo 101-8347, Japan; kumagai@kyoritsu-wu.ac.jp

**Keywords:** wheat gliadin, deamidation, peptide-bond hydrolysis, HCl treatment, cutaneous sensitization

## Abstract

Hydrochloric acid (HCl)-treated wheat protein (HWP) is widely used in various products, including foods, cosmetics and shampoos. Recently, immediate hypersensitivity towards facial soap containing HWP has been reported. HCl treatment of protein causes hydrolysis not only of main-chain amide bonds (peptide-bond hydrolysis) but also of side-chain ones (deamidation). We have already reported that gliadin, the main allergen in wheat, reduces allergenicity and increases digestibility by deamidation, indicating that deamidation and peptide-bond hydrolysis are effective to reduce the allergenicity of wheat protein. However, transdermally administered HWP is assumed to induce sensitization to orally administered wheat protein even in those who have been taking wheat products daily before sensitization. The present study was conducted to examine which structural change is responsible for the induction of cutaneous sensitization by comparing the allergenicity of deamidated and/or peptide-bond-hydrolyzed wheat gliadin. Because we have developed a deamidation method without causing peptide-bond hydrolysis, only deamidated wheat gliadin is available. Therefore, after deamidated-only, hydrolyzed-only, and deamidated and hydrolyzed gliadins were transdermally administered to mice for several weeks, the corresponding gliadin was intraperitoneally administered and allergenicity was evaluated. Transdermal administration of deamidated and hydrolyzed gliadin induced severe allergic reaction, while that of deamidated-only and hydrolyzed-only gliadin showed almost no allergic response. This result indicates that both deamidation and peptide-bond hydrolysis are necessary to increase the allergenic potency of transdermally administered wheat gliadin.

## 1. Introduction

Wheat is one of the most important cereals, not only as a carbohydrate source but also as a source of food with unique texture [1]. The unique functional properties of wheat are mostly attributed to wheat protein, which enhances the cohesiveness, viscoelasticity, extensibility, expandability, emulsifiability and foamability of the food to which it is added [1,2]. These properties of wheat protein are also useful for cosmetics such as soaps, shampoos, hair conditioners, skin moisturizers and facial creams to meet specified usages required [3]. Gluten, a protein composite of gliadin and glutenin, accounts for almost 86% of all wheat proteins, gliadin content being about 40%, and glutenin content being about 46% [4], and is often used for cosmetics after treatment with an acid like hydrochloric acid (HCl) [5]. However, despite the effectiveness of acid treatment to increase the solubility of gluten in water and improve its emulsifying and foaming properties [3], the produced peptides carry a risk of people developing an allergy to wheat when they use cosmetics containing acid-treated gluten [6].

Wheat allergy is an emerging public health problem worldwide [7] because wheat protein induces serious allergic reactions in susceptible individuals. The major allergen in wheat is gluten, which is made up of gliadin and glutenin [8, 9, 10]. Regarding patients with wheat-dependent, exercise-induced anaphylaxis (WDEIA), certain tandem sequencing sites with glutamine residues in gluten and gliadin, such as QQQPP [11], QQIPQQQ, QQLPQQQ, QQFPQQQ, QQSPEQQ, QQSPQQQ, QQYPQQQ, and PYPP [12], have been identified as the primary structure of immunoglobulin (Ig) E-binding epitopes. We have developed a technique to deamidate proteins without causing peptide-bond hydrolysis, using carboxylated cation-exchange resins [13,14,15]. We have also shown that the deamidation of glutamine resides is quite effective to reduce allergenicity of orally administered gliadin [16,17], and that oral administration of deamidated gliadin induces oral tolerance [18]. However, the mechanism of cutaneous sensitization to induce wheat allergy would be different from that of oral sensitization.

New clinical cases of wheat allergy were reported in Japan, where immediate-type allergy occurred in people who ate wheat products such as bread, noodles and cookies after using facial soap containing acid-treated gluten [19,20,21]. Because the patients had not been allergic to wheat before, those who used the facial soap were presumed to be sensitized via skin or eyes. Adachi et al. reported that HCl-treated wheat protein increased cutaneous permeability and allergic potency [22].

In cutaneous sensitization, the skin plays an important role as an interface between the external environment and immune system. The induction of adaptive cellular immunity in the skin is initiated by antigen-presenting cells such as dendritic cells, which include Langerhans cells in epidermis, mast cells, and basophils [23,24,25]. Basophils play essential roles in the skin-mediated development of the Th2 response. The invaded antigen is trapped by migrated antigen-presenting cells, which transform Th0 cells into Th2 cells. Th2 cells secrete interleukin (IL)-4, which transforms B cells into plasma cells, and then plasma cells release IgE and IgG antibodies against the antigen [26]. Following the invasion of an antigen and the establishment of the cross-linkage to IgE on the surface of mast cells, histamine is released and allergic reactions are triggered, causing symptoms such as decrease in rectal temperature [26,27]. Several findings have been reported regarding the percutaneous sensitization of mice to food allergens such as ovalbumin [28] and peanut proteins [29]. These reports have shown that cutaneous exposure to food-allergen proteins induces a Th2-type response and leads to sensitization. Food proteins/peptides are probably recognized directly by immune cells after percutaneous absorbance, which is different from intestinal sensitization in which food proteins are hydrolyzed by digestive enzymes and then recognized. Therefore, structural characteristics of allergens are crucial for recognition by immune cells, especially in the initial cutaneous sensitization process.

Acid treatment of proteins causes both peptide-bond hydrolysis and side-chain deamidation of glutamine and asparagine residues. Therefore, the reduction in molecular size by peptide-bond hydrolysis and/or side-chain deamidation of wheat gluten might be the key structural change to induce cutaneous sensitization. Because the sera from patients who used facial soap containing acid-treated gluten reacted with QPQQPFPQ in gliadin structure and its deamidated sequence PEEPFP [30], hydrolyzed and/or deamidated gliadin in gluten would be the main allergen for cutaneous sensitization.

Proteases, such as pepsin, hydrolyze proteins without causing deamidation [31,32], while carboxylated cation-exchange resins deamidate proteins without causing peptide-bond hydrolysis [13,14]. Therefore, we herein compared the cutaneous-sensitizing potency of hydrolyzed and deamidated gliadin prepared by HCl with that of hydrolyzed-only gliadin prepared by pepsin and deamidated-only gliadin prepared by carboxylated-cation-exchange resins, to identify the structural feature crucial for cutaneous sensitization. We also examined the activities of antigen-presenting cells, basophils and dendritic cells by each gliadin.

## 2. Materials and Methods

### 2.1. Wheat Protein Preparation

Wheat gliadin was extracted from gluten (Nakarai Tesque, Kyoto, Japan) with 60% ethanol. The solution was lyophilized to obtain untreated gliadin (UG). Deamidated gliadin (DG) was prepared by using cation-exchange resins of the carboxylate type as described previously [17]. Hydrolyzed gliadin (HG) was prepared by mixing UG with 0.5 mg/mL pepsin (3400 units/mg, Wako Pure Chemical Industries, Osaka, Japan) in 0.01 N HCl to a final concentration of 5 mg/mL. After incubation at 20 °C for 10 min, the enzymatic hydrolysis was quenched by adding 10 mg/mL sodium hydrogen carbonate and heating the solution at 100 °C for 5 min. The resulting solution was lyophilized to obtain HG. Hydrolyzed and deamidated gliadin (HDG) was prepared by heating 15 mg/mL UG in 0.1 N HCl at 100 °C for 1 h. The reaction was quenched by cooling and adding the same volume of 0.1 N NaOH and 0.1 M phosphate buffer at pH 7.4, and the resulting solution was lyophilized to obtain HDG.

### 2.2. Apparent Molecular Weight Distribution Measurement

The apparent molecular weight distributions for UG, DG, HG and HDG were measured using the membrane fraction method described by Hashino et al. [33]. Amicon^®^ nominal molecular weight limits of 3, 10, 50 and 100 kDa (Millipore, Billerica, MA, USA) were used as ultrafiltration membrane. Each gliadin was dissolved in water at a concentration of 1 mg/mL, and the solution was used as a feed. The protein concentration in the retentate and the permeate for each gliadin was measured using BCA protein assay kit (Thermo Fisher Scientific, Kanagawa, Japan).

### 2.3. Degree of Deamidation and Peptide-Bond Hydrolysis

The degree of deamidation was determined as the ratio of the amount of removed acid amide of DG, HG and HDG to the total acid amide of UG, as described previously [17]. Each gliadin was completely deamidated by heating in a 4 M HCl solution, and the amount of ammonia produced was measured by Conway’s micro-diffusion method [34,35]. The amount of removed acid amide was calculated by subtracting the amount of ammonia produced from DG, HG and HDG from that produced from UG. The degree of hydrolysis was measured by the method described by Kumagai et al. [13]. The degree of hydrolysis was expressed as the proportion of nitrogen contained in the 0.6 M trichloroacetic acid-soluble fraction to the total nitrogen content.

### 2.4. Animals

Seven-week-old female BALB/c mice were purchased from Japan SLC, Inc., (Shizuoka, Japan). All animal experiments were performed following the Guidelines for Animal Experiments of the College of Bioresource Sciences of Nihon University (approval number: AP14B003, AP15B017, and AP15B028), which meet the ethical guidelines for experimental animals in Japan. Water was made available from bottles, and a commercial mouse diet (CLEA Rodent Diet CE-2, CLEA Japan, Inc., Tokyo, Japan) was provided ad libitum.

### 2.5. Transdermal Sensitization

**Figure 1:**
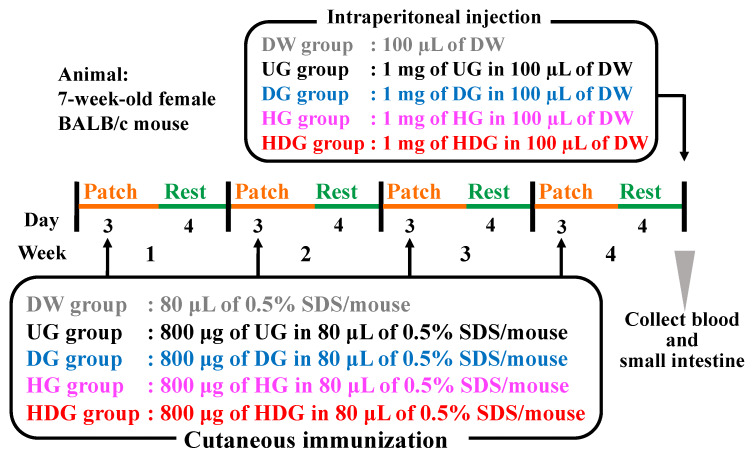
Experimental design to evaluate allergenicity and cutaneous sensitivity of each gliadin (n = 8).

Transdermal sensitization was performed according to the method proposed by Adachi et al. [22] As illustrated in Figure 1, the mice were divided into five groups (n = 8): the distilled water (DW) group was patched with 80 μL of DW containing 0.5% (*w*/*v*) SDS to be unsensitized; the UG, DG, HG, and HDG groups were sensitized with 800 μg of the corresponding gliadin in 80 μL of DW containing 0.5% (*w*/*v*) SDS. The stratum corneum of the dorsal shaved skin of mice was removed by tape-stripping, repeated 20 times before sensitization. The gliadin suspension was applied to the shaved area of each mouse with a patch (CARELEAVESTM, Nichiban Co., Ltd., Tokyo, Japan), and the patched area was covered with surgical tape. The mice were exposed to each antigen for 3 days and were then rested for the next 4 days by removing the patch. Transdermal exposure to antigen was repeated for four cycles.

### 2.6. Systemic Anaphylaxis Induction

The transdermally sensitized mice were intraperitoneally challenged with 1 mg of antigen in 100 μL of DW 30 min on the 28th day after the final sensitization cycle.

The rectal temperature of mice was measured with a rectal thermometer (SK-1260, Sato Keiryoki Mfg Co., Ltd., Tokyo, Japan) 30 min after intraperitoneal challenge.

The anaphylactic response to the antigen-challenged mice was evaluated by scoring symptoms at the same time. The anaphylactic scores were categorized into the following six levels: Level 0, no symptoms; level 1, scratching and rubbing around the snout and head; level 2, showing puffiness around the eyes and snout, pilar erection, reduced activity or standing still with an increased respiratory rate; level 3, motionless for more than 1 min, transient convulsion, wheezing, labored respiration, or lying prone on stomach; level 4, no response to whisker stimuli, slight or no activity after prodding, tremor, convulsion, or loss of consciousness; level 5, death.

### 2.7. Measurement of Serum Histamine and Gliadin Specific IgE and IgG1 Levels

The blood was collected from the postcaval vein under anesthesia after the measurement of rectal temperature and anaphylactic scores. The blood samples for the measurement of gliadin specific IgE and IgG1 levels were centrifuged at 1300× *g* and 20 °C for 15 min to give the sera, which were stored at −80 °C until assay. The levels of gliadin-specific IgE and IgG1 in the sera were determined as described previously [17]. For the measurement of histamine levels, blood from individual mouse was collected into 0.5-mL heparinized tubes. The amount of histamine in the plasma was measured by high-performance liquid chromatography according to the on-column conversion method by using *o*-phthalaldehyde according to the manufacturer’s instruction.

### 2.8. Measurement of Mcpt8 Expression Level

Skin biopsy specimens of the sensitized site were obtained after blood collection. To extract RNA and measure Mcpt8 expression level, the specimens were homogenized in Trizol (GIBCO BRL, Grand Island, NY, USA) using a Polytron (NS-52K, Microtec Co., Ltd., Chiba, Japan) and RNA was reverse transcribed into cDNA using a thermal cycler (MJ Research, Watertown, MA, USA).

### 2.9. In Vitro Skin Sensitization Test

To evaluate the immunological response of skin macrophage to HG and HDG, the human cell-line activation test (h-CLAT) was conducted according to the method described by Ashikage et al. [36]. Briefly, THP-1 cells from ATCC (Manassas, VA, USA) were cultured in RPMI-1640 (Sigma-Aldrich, Dorset, UK) supplemented with 10% fetal bovine serum, 100 units/mL penicillin, and 100 μg/mL streptomycin (Wako Pure Chemical) at 37 °C under 5% CO_2_ in 12-well plates (1.0 × 10^6^ cells/mL/well) with 1.0 mg/mL HG or HDG for 18 h. In the control group, HG and HDG were not added. Thereafter, the cells were washed twice with phosphate-buffered saline containing 0.1% bovine serum albumin. Next, total RNA was extracted from the cells.

### 2.10. Quantitative Real-Time PCR

The total RNA extracted from mouse cutaneous cells and THP-1 cells were reverse-transcribed into cDNA using a thermal cycler TP350 (Takara Bio, Shiga, Japan). Real-time PCR was performed on CFX96 (BIO-RAD, Hercules, CA, USA) and SYBR green PCR mix (Applied Biosystems, Cheshire, UK). The results were expressed as a copy number ratio of the target mRNA to glyceraldehyde 3-phosphate dehydrogenase (GAPDH) mRNA. The primers used in PCR were as follows; forward primer for GAPDH: 5′-CGTCTTCACCACCATGGAGA-3′, reverse primer for GAPDH: 5′-CGGCCATCACGCCACAGTTT-3′, forward primer for Mcpt8: 5′-CCGGAATTCATGTTCCTGCTCCTGGTCC-3′, reverse primer for Mcpt8: 5′-CGCGGATCCCTAGGGTTGTTGCAGGAGTTTCATTG-3′, forward primer for CD54: 5′-AGGCCACCCCAGAGGACAAC-3′, reverse primer for CD54: 5′-TGACTGCGGCTGCTACCACA-3′; forward primer for CD86: 5′-TGGTCAGGGAGGGGTTTTGG-3′, reverse primer for CD86: 5′-GCCCCGGGTGATCTGTGTCT-3′.

### 2.11. Statistical Analysis

Statistical analyses were performed using the Mac Toukeikaiseki Version 3.0 software package (Esumi, Co., Ltd., Tokyo, Japan). All data were expressed as mean ± SE, and the significance of the differences (*p* values) among groups was evaluated by one-way ANOVA followed by a Tukey–Kramer test.

## 3. Results

### 3.1. Degrees of Deamidation and/or Peptide-Bond Hydrolysis of Wheat Gliadins

**Figure 2:**
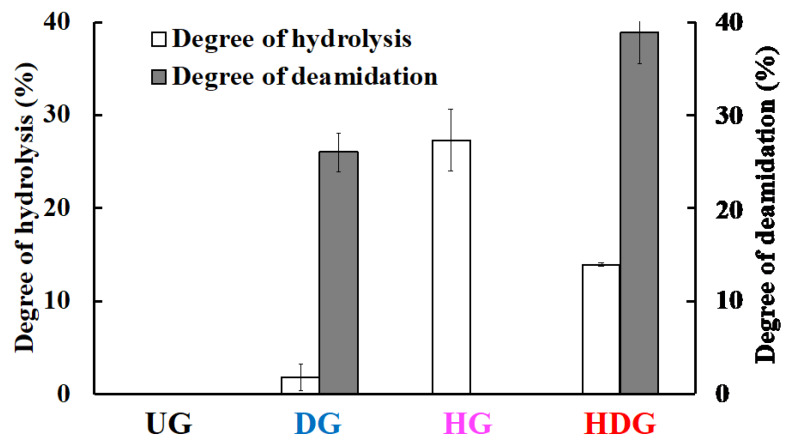
Degrees of deamidation and peptide-bond hydrolysis of wheat gliadins. Each value is the mean of four experiments with SE shown as a vertical bar.

**Figure 3:**
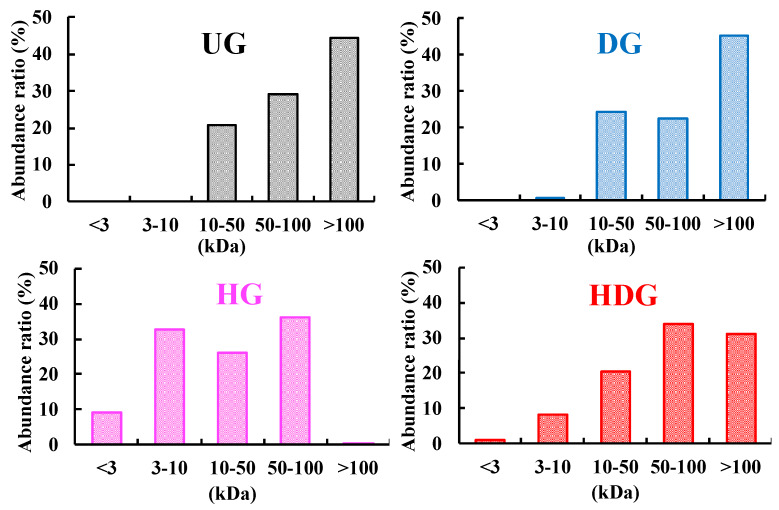
Molecular weight distribution of gliadins.

The degrees of deamidation of UG, DG, HG, and HDG were 0%, 26%, 0%, and 39%, respectively, while those of peptide-bond hydrolysis of UG, DG, HG, and HDG were 0%, <1%, 26%, and 13%, respectively (Figure 2). Figure 3 shows the apparent molecular weight distributions for each gliadin, measured by the ultrafiltration method. The abundance ratios of peptides with molecular weights lower than 10 kDa in UG, DG, HG, and HDG were 0%, <1%, 42%, and 9%, respectively. This distribution was consistent with the order of degree of peptide-bond hydrolysis of gliadins. The abundance ratios of peptides with molecular weight higher than 100 kDa in UG, DG, HG, and HDG were 44%, 45%, 0%, and 31%, respectively, which was consistent with the inverse order of degree of peptide-bond hydrolysis of gliadins. HDG possessed both high- (>100 kDa) and low- (<10 kDa) molecular-weight peptides and the range of molecular weight was widely distributed.

### 3.2. Cutaneous Sensitivity of Deamidated and/or Peptide-Bond-Hydrolyzed Gliadin

**Figure 4:**
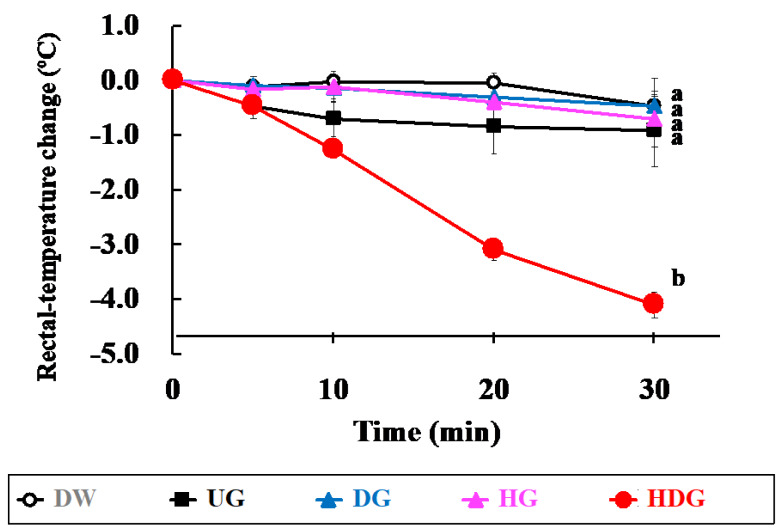
Change in the rectal temperature of mice after intraperitoneal challenge of each gliadin followed by cutaneous sensitization with the corresponding gliadin. Values with different letters at 30 min are significantly different at *p* < 0.01 based on the Tukey–Kramer test. Each value is the mean of eight experiments with SE shown as a vertical bar.

**Figure 5:**
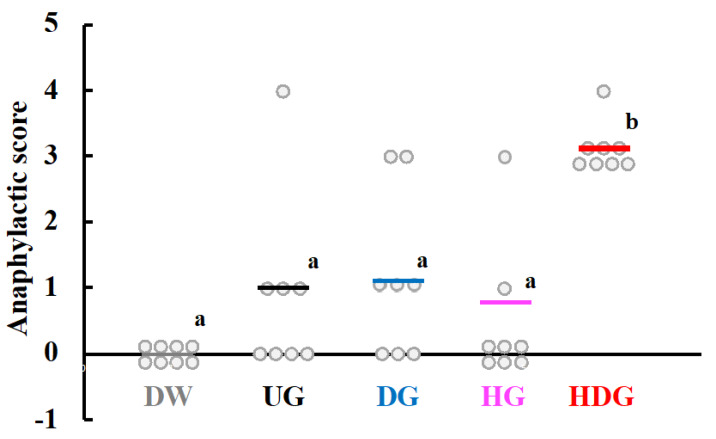
Anaphylactic scores of mice after intraperitoneal challenge of gliadin followed by cutaneous sensitization with the corresponding gliadin. Values with different letters are significantly different at *p* < 0.05 based on the Tukey–Kramer test. The bars represent the average score for each group (n = 8).

**Figure 6:**
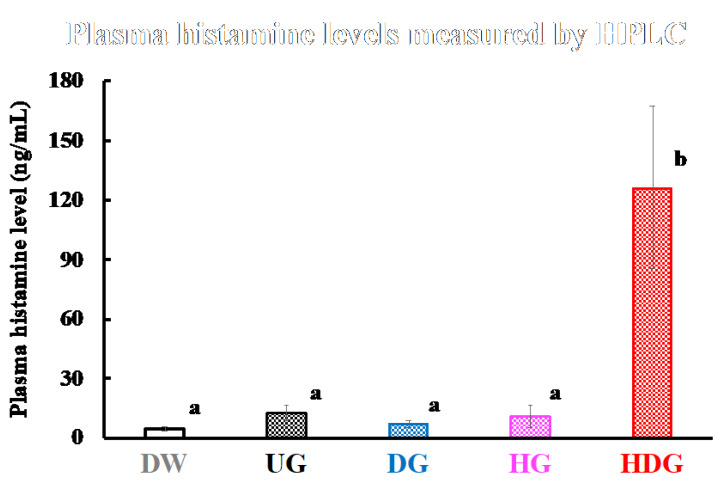
Plasma histamine levels of mice after intraperitoneal challenge of gliadin followed by cutaneous sensitization with the corresponding gliadin. Values with different letters are significantly different at *p* < 0.05 based on the Tukey–Kramer test. Each value is the mean of eight experiments with SE shown as a vertical bar.

Figure 4 shows the change in rectal temperature of mice 30 min after intraperitoneal challenge. The average rectal temperature of HDG-sensitized mice decreased by approximately 4.2 °C for 30 min, while the average rectal temperature of UG-, DG- and HG-sensitized mice was at the same level as that of unsensitized mice (DW). The rectal temperature of HDG-sensitized mice at 30 min was significantly lower than that of the other groups at the same time point (*p* < 0.01). After four transdermal sensitizations of each gliadin, immediate hypersensitivity reactions were examined by intraperitoneal challenge of the same antigens used for the sensitization. Figure 5 shows the anaphylactic scores of these mice. The DW group showed no symptoms, with an average score of 0, while DG- and HG-sensitized mice presented moderate symptoms, with an average score of 1. Severe symptoms were observed in HDG-sensitized mice, with an average score of 4. The average score of the HDG group was significantly higher than that of the other groups (*p* < 0.05). Figure 6 shows the plasma histamine levels of mice after intraperitoneal challenge with each gliadin. The mean value of plasma histamine level of HDG-sensitized mice was significantly higher than that of UG-, DG- and HG-sensitized mice and unsensitized mice (*p* < 0.05).

### 3.3. Gliadin-Specific Immunoglobulin Levels

**Figure 7:**
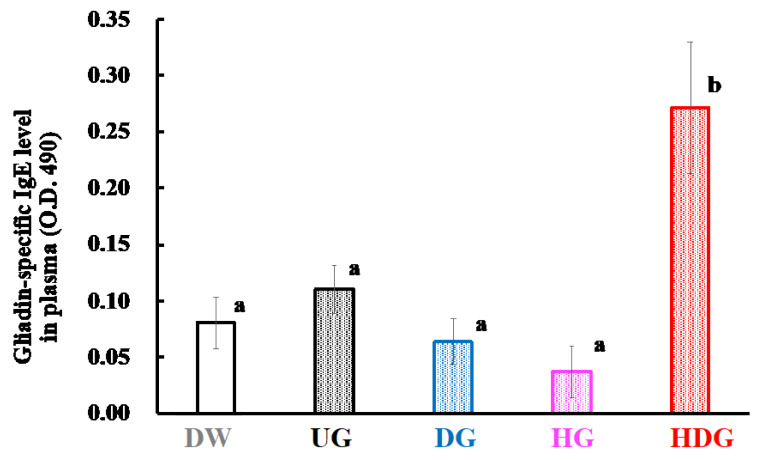
Gliadin-specific IgE levels in the plasma of mice after intraperitoneal challenge of gliadin followed by cutaneous sensitization with the corresponding gliadin. Values with different letters are significantly different at *p* < 0.05 based on the Tukey–Kramer test. Each value is the mean of eight experiments with SE shown as a vertical bar.

**Figure 8:**
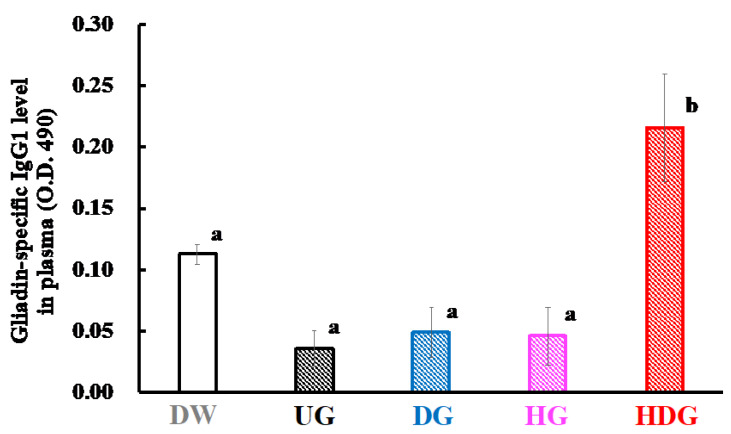
Gliadin-specific IgG1 level in the plasma of mice after intraperitoneal challenge of gliadin followed by cutaneous sensitization with the corresponding gliadin. Values with different letters are significantly different at *p* < 0.05 based on the Tukey–Kramer test. Each value is the mean of eight experiments with SE shown as a vertical bar.

Figure 7 and Figure 8 show the levels of IgE and IgG1 specific for native gliadin in the sera of the UG-, DG- HG- and HDG-sensitized mice. The IgE and IgG1 levels of HDG-sensitized mice were significantly increased compared with those of unsensitized mice (*p* < 0.05). On the other hand, no significant difference in the gliadin-specific IgE and IgG1 levels in the sera was observed in the UG-, DG- and HG-sensitized mice and unsensitized mice.

### 3.4. mRNA Level of Mcpt8 at the Skin Site

**Figure 9:**
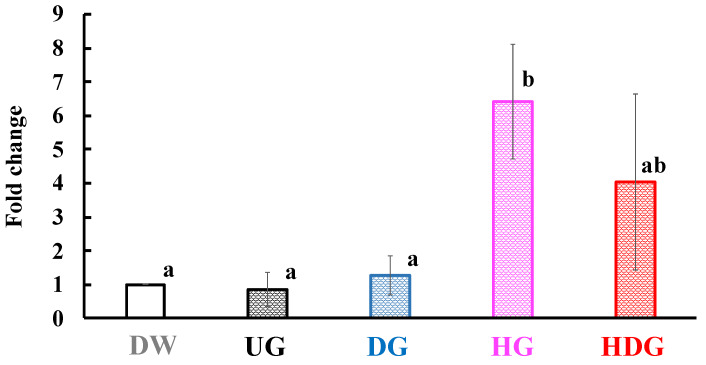
Mcpt8 mRNA expression level in the skin of mice after intraperitoneal challenge of gliadin followed by cutaneous sensitization with the corresponding gliadin. Values with different letters are significantly different at *p* < 0.05 based on the Tukey–Kramer test. Each value is the mean of eight experiments with SE shown as a vertical bar.

To investigate the migration of basophils at the skin site after antigen exposure, we examined the expression level of Mcpt8, a basophil maker. Figure 9 shows mRNA expression level of Mcpt8 at the skin site after final transdermal sensitization with UG, DG, HG and HDG. The Mcpt8 mRNA expression level increased in the HG and HDG groups but not in the other groups.

### 3.5. mRNA Levels of CD54 and CD86 in THP-1 Cells

**Figure 10:**
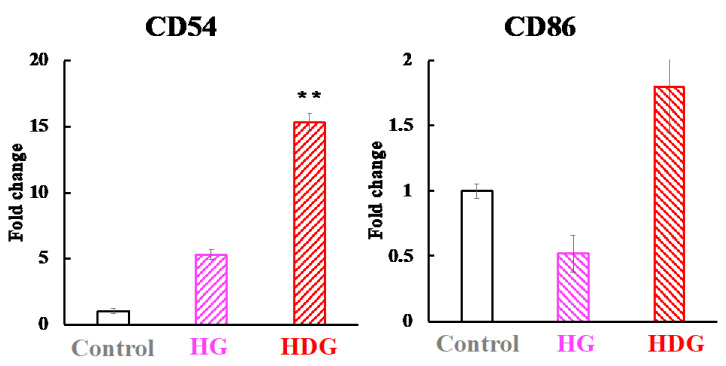
CD54 and CD86 mRNA expression levels in THP-1 cells after HDG or HG exposure in the h-CLAT method. The value with two asterisks is significantly different from that of the DW group at *p* < 0.01 by the Tukey test. Each value is the mean of four experiments, with SE shown as a vertical bar.

Figure 10 shows the CD54 and CD86 mRNA expression levels in the THP-1 cells after HDG or HG exposure in the h-CLAT method. The mRNA expression levels of CD54 in the HG and HDG group were 5.6- and 15.6-fold higher than that in the DW group, respectively. The mRNA expression level of CD86 in the HDG group was 1.8-fold higher than that in the DW group, while the mRNA expression level of CD86 in the HG group was not increased.

## 4. Discussion

HCl-treated wheat protein had increased cutaneous permeability and allergic potency. This treatment of proteins causes both peptide-bond hydrolysis and side-chain deamidation. Therefore, we focused on peptide-bond hydrolysis and side-chain deamidation of wheat gluten, which might be the key structural change to induce cutaneous sensitization. We prepared UG, DG, HG, and HDG from gluten to clarify the structural feature responsible for the cutaneous sensitization process. HCl treatment of gliadin afforded HDG with a 13% degree of peptide-bond hydrolysis and a 39% degree of side-chain deamidation. Enzyme and cation-exchange resins selectively hydrolyzed peptide bonds and side-chain amide bonds, respectively. Enzymatic treatment of gliadin produced HG with a 26% degree of peptide-bond hydrolysis and a 0% degree of side-chain deamidation, while cation-exchange-resin treatment produced DG with a <1% degree of peptide-bond hydrolysis and a 26% degree of side-chain deamidation. These results indicate that deamidated-only gliadin, hydrolyzed-only gliadin, and hydrolyzed and deamidated gliadin could be prepared.

Using each gliadin, we evaluated the allergenicity after cutaneous immunization followed by intraperitoneal injection of gliadin. The anaphylactic score in the HDG group was about 3, which was significantly higher than that in the other groups (*p* < 0.05). Although the UG, DG, and HG groups showed slight anaphylactic symptoms after intraperitoneal injection of each gliadin, scoring about 1, no significant difference was found to the DW group. In addition, the rectal temperature of the DW, UG, DG, and HG groups was retained at almost the same level for 30 min, while that of the HDG group dropped by about 4.2 °C after 30 min, showing significant difference from the other groups (*p* < 0.01). The severe allergic responses observed in the HDG group were in good accordance with those reported in the literatures using HCl-treated wheat protein [37]. Therefore, the sharp contrast between the HDG and other groups regarding allergic reactions demonstrated that both peptide-bond hydrolysis and side-chain deamidation are crucial for cutaneous sensitization. In other words, our results indicate that DG and HG have a lower risk of inducing allergic reactions after cutaneous immunization compared with HDG.

The severe allergic reactions observed only in the HDG group could be attributed to the high sensitizing potency of HDG. The plasma histamine, gliadin-specific IgE, and IgG1 levels in the HDG group were significantly higher than those in the other groups (*p* < 0.05), indicating that mice in the HDG group were sensitized with HDG and induced a Th2-immune response. However, the IgE and IgG1 levels specific to native gliadin in the UG, DG and HG groups were significantly lower than those in the HDG group and were at similar levels to those in the DW group. These results suggest that UG, DG and HG have low sensitizing abilities.

The difference in sensitizing abilities among the gliadins can be explained by the activities of antigen-presenting cells. The immune process for cutaneous sensitization begins with antigen presentation by cells such as dendritic cells and basophils. Dendritic cells such as Langerhans cells and basophils play important roles for Th2 induction and IgE production upon epicutaneous protein exposure [23]. Basophils lead to Th2 skewing, supporting dendritic-cell induction of the Th2 response upon antigen-protein exposure by trogocytosis of peptide–major histocompatibility class II complexes [25,38]. In addition, basophils directly stimulate T cells and promote IL-4 production in CD4^+^ T cells upon exposure to peptide antigens [25]. The enhancement of basophils in HWP-sensitized patients was reported to be induced by HWP administration [39]. Moreover, HCl-treated gliadin increased degradation by bone marrow-derived dendritic cells [40]. We evaluated the sensitizing property of each gliadin by measuring the activities of basophils and dendritic cells. The mRNA expression level of Mcpt8 under the skin was higher in the HG and HDG groups than in the DW, UG and DG groups immediately after intraperitoneal administration of each gliadin. This finding indicates that HG and HDG were recognized as antigens by basophils, but UG and DG were not recognized and the sensitizing abilities of UG and DG were lower than those of HG and HDG. Migration of basophils is not sufficient to explain the sensitization potency of HDG because HG and HDG induced migration of basophils. However, it is difficult to isolate dendritic cells from mouse skin without activating them. Therefore, we evaluated the activity of dendritic cells in vitro after the addition of HDG or HG to THP-1 cells by the h-CLAT method [41], measuring the mRNA levels of CD54 and 86. The mRNA levels of CD54 and 86 in the HDG group increased, but little or no increase was observed in the HG group. These results indicate that dual activation of dendritic cells and basophils is required for the highly sensitizing and allergic potency of HDG.

This study clarified that both peptide-bond hydrolysis and deamidation of gliadin are necessary to induce high cutaneous sensitization. Peptide-bond hydrolysis reduces the molecular weight of the allergen protein, increasing its permeability through the skin barrier. The invaded antigen is then captured by antigen-presenting cells. Different from HDG, HG activated basophils but rarely induced allergic reactions after cutaneous immunization followed by intraperitoneal injection of HG. Therefore, the glutamic acid residues in gliadin may play an important role in activating dendritic cells and inducing allergic reactions after permeation through skin.

Although we did not examine the crucial limit of the molecular size of gliadin for skin permeability using gliadins with different degrees of hydrolysis, we showed that gliadin has to be hydrolyzed to permeate through skin. Deamidation using cation-exchange resins does not hydrolyze peptide bonds, which means DG cannot permeate through skin. In addition, as shown in our previous paper, DG has high foaming capacity, the value of which is higher than that of egg white protein [15]. Therefore, DG could be used as a safe foaming agent both in foods and cosmetics.

## 5. Conclusions

We have revealed that both peptide-bond hydrolysis and side-chain deamidation of gliadin are necessary for cutaneous sensitization, indicating that both the reduction in molecular size and increase in glutamic-acid residues are crucial for activating antigen-presenting cells and inducing allergic reactions. Our study has also shown that deamidated gliadin with no peptide-bond hydrolysis (DG) is promising as an alternative material for cosmetics because deamidation improves physicochemical properties such as foamability and emulsifiability of gliadin and DG scarcely activates antigen-presenting cells and shows no allergic response after transdermal exposure.
